# Comparison of variable and model selection methods for genetic association studies using the GAW15 simulated data

**DOI:** 10.1186/1753-6561-1-s1-s34

**Published:** 2007-12-18

**Authors:** Zhan Ye, Elizabeth J Atkinson, Brooke L Fridley, Mariza de Andrade

**Affiliations:** 1 Department of Mathematical Sciences, Michigan Technological University, 1400 Townsend Drive, Houghton, Michigan 49931, USA; 2 Divison of Biostatistics, Mayo Clinic College of Medicine, 200 First Street SW, Harwick 7, Rochester, Minnesota 55905, USA

## Abstract

We compared and evaluated several variable and model selection methods using Bayesian and non-Bayesian approaches for three replicates of the Genetic Analysis Workshop 15 (GAW15) simulated data. In doing so, two phenotypes were utilized: rheumatoid arthritis (RA) affection status as a binary trait and IgM as a continuous measure. The analyses were performed adjusting for sex, age, and smoking status. For both outcomes, all the methods were comparable in finding the single-nucleotide polymorphisms (SNPs) generated to have a genetic signal. We successfully identified the susceptibility SNPs for RA in the HLA region (chromosome 6), and chromosome 18, and the susceptibility SNP for IgM on chromosome 11; however, many of the methods produced false-positive results.

The answers to Problem 3 were requested and known to the authors.

## Background

Variable and model approaches are becoming increasing important due to the advances in DNA chip technology, resulting in as many as 500,000 single-nucleotide polymorphisms (SNPs). However, it is unclear when a particular statistical method should be used, and how Bayesian methods compare with more standard frequentist approaches. The goal of this paper is to compare different methods using Bayesian and non-Bayesian approaches for binary and continuous outcomes. We used three replication sets from the Genetic Analysis Workshop (GAW15) simulated data and focused on chromosomes 6 and 18 for rheumatoid arthritis (RA) affection status, and on chromosome 11 for the continuous outcome, IgM. In addition to chromosomes 6, 11, and 18, chromosome 19 was used as a control (null).

The data were simulated to have a signal at 115.28 cM (SNPs 389–394) on chromosome 11 with the continuous outcome, IgM, at 49.46 cM (SNPs 152–154) on chromosome 6 with RA affection status, and at 94.27 cM (SNPs 267–270) with a controlled effect of DR on anti-CCp and an increased risk on RA on chromosome 18.

For the continuous phenotype, we used the data from affected sib pairs, since IgM was observed only on the cases. Thus, we divided the data in two data sets, training and testing. The training set consisted of one of the affected siblings from each pedigree chosen at random, and the testing set consisted of the remaining affected siblings. For the binary analysis, the RA case was one of the affected siblings, from the 1500 simulated affected sib pairs, and the RA case was selected such that the percentages of males and females between the RA siblings and the unrelated controls (2000 controls overall) were similar (i.e., frequency matched on sex). Furthermore, covariates of age, sex, and smoking status were also available for all individuals. Overall, 1500 RA cases and 2000 controls were used to analyze 674, 303, 93 SNPs on chromosome 6, 18, and 19, respectively. One thousand five hundred affected pairs were selected for the IgM analysis using 492 SNPs on chromosome 11.

## Methods

### Single-marker test and stepwise variable selection

We investigated five variable and model selection strategies. The first strategy was to perform single SNP analysis. For IgM and RA affection status, standard linear regression and logistic regression models were fit including age, sex, smoking status, and the SNP genotype as covariates. We used an alpha level of 0.01 to determine variable significance. The second strategy was to perform stepwise variable selection to build a multivariable model for each chromosome separately. A cutoff of 0.01 was used for variable selection (i.e., for a variable to enter or stay in the model).

### Rpart and random forest methods

The third and fourth strategies were tree-related. Classification tree uses a recursive partitioning tree approach with splitting and pruning rules [1]. Splitting rules are used to examine all possible splits of the full group of subjects and to identify the variable at each level that produces the most homogenous children. All trees were fit using the R library *rpart *[2]. We trimmed the trees to include only splits that improved the overall model by at least 2% and listed any of the main variables in those trees in our comparison tables. Random forest (RF) is an expansion of the tree concept, where thousands of trees are grown and averaged together. Each tree is an independent bootstrap sample of the data, and at each node *m *variables are randomly selected out of all *M *possible variables. The result of this averaging is a summary of each variable's overall importance. This approach also provides an overall measure of how well we can expect to accurately predict our endpoints. All models were fit using the R library *randomForest *[3]. A subset of high-interest SNPs was formed including the union of the top five variables that separated out the cases, the top five that separated out the controls, the top five overall using the accuracy measure, and the top five overall using the Gini index criteria (for case and control samples).

### Bayesian model averaging approach

The last strategy implemented was Bayesian model averaging (BMA). BMA is a Bayesian method designed to account for model uncertainty and to propagate this uncertainty through the analysis to the inferences. The BMA method produces a posterior probability for each possible model in addition to the posterior probability for each predictor (i.e., SNP). That is, the BMA posterior distribution of Δ, where Δ represents the parameter, is

$$p(\Delta |D)=\sum_{k=1}^{K}p(\Delta |D,M_{k})p(M_{k}|D),$$

where *p*(Δ|*D*, *M*_*k*_) is the posterior distribution of Δ given the model *M*_*k*_, and *p*(*M*_*k*_|*D*) is the posterior probability that *M*_*k *_is the correct model, given that one of the models considered is correct and data *D *[4]. Because the method fits all possible models, the BMA method is limited in terms of the number of predictors that can be included in the model.

The BMA method was fit on the union of the SNPs from the random forest method (top 25 SNPs for the IgM analysis and top 10 SNPs for the RA status analysis). Once the BMA analysis was fit to the "top SNPs", a variable was selected if the posterior probability for the particular variable was greater than 0.80. The analyses were performed using the R library BMA [5].

## Results

Tables 1 and 2 display the results from the analyses on chromosomes 6 and 18 for RA affection status. Most of the methods detected the genetic signal for all three replicates. Some of the methods detected SNPs close to the simulated associated SNPs, which may be due to high linkage disequilibrium (LD) level between these sets of SNPs.

**Table 1:** Variables selected from each of the methods for RA status and chromosome 6^a^

	Replication	SNPs/bp				
		
Methods	base pair	*SNP152*/*32447150*^b^	*SNP153*/*32499470*	*SNP154*/*32521280*	SNP155/32772270	SNP162/37363880
Single marker test^a^	Rep50	X	X	X	X	X
	Rep51	X	X	X	X	X
	Rep52	X	X	X	X	X
						
Random forest	Rep50	X	X	X	X	-
	Rep51	X	X	X	X	X
	Rep52	X	X	X	X	X
						
Classification tree^c^	Rep50	X	X	X	X	-
	Rep51	X	X	X	X	X
	Rep52	X	X	X	X	X
						
Stepwise regression	Rep50	X	X	X	X	X
	Rep51	X	X	X	X	X
	Rep52	-	X	X	-	-
						
BMA	Rep50	X	X	X	X	-
	Rep51	X	X	X	X	X
	Rep52	-	X	X	-	X

^a^We analyzed 1500 cases, 200 controls, and 647 SNPs.

^b^Italics indicates SNPs simulated with a genetic signal.

^c^Classification tree method implemented using rpart.

**Table 2:** Variables selected from each of the methods for RA status andchromosome 18^a^

	Replication	SNPs/bp				
		
Methods	base pair	SNP265/65345780	SNP266/65694950	*SNP268*/*66045170*^b^	*SNP269*/*66048930*	SNP273/6721130
Single marker test^a^	Rep50	-	X	X	X	X
	Rep51	-	-	X	X	-
	Rep52	-	-	X	X	-
						
Random forest	Rep50	-	-	-	X	-
	Rep51	-	-	-	X	-
	Rep52	-	X	X	X	-
						
Classification tree^c^	Rep50	-	X	X	X	-
	Rep51	-	-	X	X	-
	Rep52	-	-	X	X	-
						
Stepwise regression	Rep50	-	-	X	X	-
	Rep51	-	-	X	X	-
	Rep52	-	-	X	X	-
						
BMA	Rep50	X	-	X	X	-
	Rep51	-	-	-	X	-
	Rep52	-	-	X	X	-

^a^We analyzed 1500 cases, 2000 controls, and 303 SNPs.

^b^Italics indicates SNPs simulated with a genetic signal.

^c^Classification tree method implemented using rpart.

Additional file 1 shows the results for the IgM analysis on chromosome 11 for the training and testing data sets. SNP389 was picked up by every method in all three replicates and in both sets except for one replicate (52) in the training set. In addition, only SNP387 was selected by the single-SNP and random forest strategies for all three replicates. No other SNP was consistently selected using different strategies. Selection of the SNP by the single SNP and random forest methods may be an artifact of LD between two close SNPs (high LD).

Lastly, we ran every method for RA affection status on chromosome 19 (our false-positive control). No significant results were observed for any of the methods except for the single-SNP analysis. SNPs 74 and 10 were picked up (*p *< 0.01) in Replicates 50 and 51, respectively, and SNPs 38, 39, and 79 in Replicate 52 (*p *< 0.01).

## Discussion

We have presented a variety of strategies for variable selection for binary and continuous outcomes. We also compared these strategies using three replicate data sets from the simulation data for chromosome 11 using IgM and chromosomes 6, 18, and 19 using RA affection status. All of these methods identified the genomic region correctly. However, we cannot conclude whether there is a "best" strategy since most, if not all, of the methods were able to pick the correct SNPs. It is difficult to compare the different strategies because different criteria and assumptions were used in the various approaches. For instance, BMA analysis was performed on a subset of SNPs due to the restrictions on the number of variables BMA could handle. The classification tree and random forest approaches considered interactions. Single-SNP tests and stepwise regression analysis looked for additive effects of the SNPs. BMA and stepwise regression looked for important variables to identify the best overall model. Thus, the method of choice should be based on the goal of the analysis. The success of different approaches will necessarily depend on issues such as single versus multiple SNPs, additive versus dominant or recessive effects, and main effects versus interactions.

## Conclusion

Regression methods and Bayesian methods correctly identify the target loci in the simulated data. However, the advantages of these methods could not be exploited due to the underlying simulation model, i.e., the strong signal of causal SNPs, and the pre-selected criteria of SNPs.

## Competing interests

The author(s) declare that they have no competing interests.

## Supplementary Material

Additional file 1 Variables selected from each of the methods for IgM phenotype and chromosome 11 Click here for file
