# Supplementary material for: Comparison of variable and model selection methods for genetic association studies using the GAW15 simulated data
Source: BMC Proc. 2007 Dec 18;1(Suppl 1):S34. doi: 10.1186/1753-6561-1-s1-s34 (PMC2367491; doi:10.1186/1753-6561-1-s1-s34)
Supplement: Additional file 1 — Variables selected from each of the methods for IgM phenotype and chromosome 11 [file 1753-6561-1-S1-S34-S1.doc]

**Variables selected from each of the methods for IgM phenotype and chromosome 11**

|  |  | SNP | SNP | SNP | SNP | SNP | SNP | SNP | SNP | SNP | SNP | SNP | SNP | SNP | *SNP* | SNP | SNP | SNP |
| --- | --- | --- | --- | --- | --- | --- | --- | --- | --- | --- | --- | --- | --- | --- | --- | --- | --- | --- |
|  |  | 139 | 142 | 166 | 168 | 175 | 206 | 208 | 216 | 295 | 307 | 335 | 386 | 387 | *389*^a^ | 395 | 396 | 399 |
| **Training data sets (1500 individuals, 492 markers)** | | | | | | | | | | | | | | | | | | |
| Single marker test ^a^ | Rep50 | - | - | - | - | - | - | - | - | X | - | X | - | X | X | X | - | - |
|  | Rep51 | - | - | - | - | - | - | - | - | - | - | - | - | X | X | - | - | - |
|  | Rep52 | - | - | - | - | - | - | - | - | - | - | X | - | X | - | X | - | - |
|  |  |  |  |  |  |  |  |  |  |  |  |  |  |  |  |  |  |  |
| Random Forest | Rep50 | - | - | - | - | - | - | - | - | - | - | - | - | X | X | - | - | - |
|  | Rep51 | - | - | - | - | - | - | - | - | - | - | - | - | X | X | - | - | - |
|  | Rep52 | - | - | - | - | - | - | - | - | - | - | - | X | X | X | - | X | - |
|  |  |  |  |  |  |  |  |  |  |  |  |  |  |  |  |  |  |  |
| Classification tree ^b^ | Rep50 | - | - | - | - | - | - | - | X | - | - | - | - | - | X | - | - | - |
|  | Rep51 | - | - | - | - | - | - | - | - | - | - | - | - | - | X | - | - | - |
|  | Rep52 | - | - | - | - | - | - | - | - | - | - | - | - | - | X | - | - | - |
|  |  |  |  |  |  |  |  |  |  |  |  |  |  |  |  |  |  |  |
| Stepwise regression | Rep50 | - | - | - | - | - | - | X | - | X | - | - | - | - | X | - | - | - |
|  | Rep51 | - | - | - | - | - | - | - | - | - | - | - | - | - | X | - | - | - |
|  | Rep52 | - | - | - | - | - | - | - | - | - | - | - | - | - | X | - | - | - |
|  |  |  |  |  |  |  |  |  |  |  |  |  |  |  |  |  |  |  |
| BMA | Rep50 | - | - | - | X | X | - | - | - | X | - | - | - | - | X | - | - | - |
|  | Rep51 | - | - | - | - | - | - | - | - | - | - | - | - | - | X | - | - | - |
|  | Rep52 | - | - | - | - | - | - | - | - | - | - | - | - | - | X | - | - | - |
|  |  |  |  |  |  |  |  |  |  |  |  |  |  |  |  |  |  |  |
| **Testing data sets (1500 individuals, 492 markers)** | | | | | | | | | | | | | | | | | | |
| Single marker test ^a^ | Rep50 | - | - | - | - | - | - | - | - | - | - | - | - | X | X | - | - | X |
|  | Rep51 | - | - | - | - | - | X | - | - | - | - | - | - | X | X | - | - | X |
|  | Rep52 | - | - | - | - | - | - | - | - | - | X | - | - | X | X | - | - | X |
|  |  |  |  |  |  |  |  |  |  |  |  |  |  |  |  |  |  |  |
| Random forest | Rep50 | - | - | - | - | - | - | - | - | - | - | - | X | - | X | - | - | - |
|  | Rep51 | - | - | - | - | - | - | - | - | - | - | - | - | X | X | - | - | - |
|  | Rep52 | - | - | - | - | - | - | - | - | - | - | - | - | X | X | - | - | - |
|  |  |  |  |  |  |  |  |  |  |  |  |  |  |  |  |  |  |  |
| Classification tree ^b^ | Rep50 | - | - | - | - | - | - | - | - | - | - | - | - | - | X | X | - | - |
|  | Rep51 | - | - | X | - | - | - | - | - | - | - | - | - | - | X | - | - | - |
|  | Rep52 | - | - | - | - | - | - | - | - | - | - | - | - | - | X | - | - | - |
|  |  |  |  |  |  |  |  |  |  |  |  |  |  |  |  |  |  |  |
| Stepwise regression | Rep50 | - | - | - | - | - | - | - | - | - | - | - | - | - | X | - | - | - |
|  | Rep51 | - | - | - | - | - | - | - | - | - | - | - | - | - | X | - | - | - |
|  | Rep52 | - | - | - | - | - | - | - | - | - | - | - | - | - | X | - | - | - |
|  |  |  |  |  |  |  |  |  |  |  |  |  |  |  |  |  |  |  |
| BMA | Rep50 | X | - | - | - | - | - | - | - | - | - | - | - | - | X | - | - | - |
|  | Rep51 | - | - | - | - | - | - | - | - | - | - | - | - | - | X | - | - | - |
|  | Rep52 | - | X | - | - | - | - | - | - | - | - | - | - | - | X | - | - | - |

^a^Italics indicates SNPs simulated with a genetic signal.

^b^Classification tree method implemented using rpart.
